# Anatomical Variations of the External Jugular Vein: A Pictorial and Critical Review

**DOI:** 10.3390/medicina59030622

**Published:** 2023-03-21

**Authors:** Mugurel Constantin Rusu, Răzvan Costin Tudose, Alexandra Diana Vrapciu, Corneliu Toader, Şerban Arghir Popescu

**Affiliations:** 1 Division of Anatomy, Faculty of Stomatology, “Carol Davila” University of Medicine and Pharmacy, 020021 Bucharest, Romania; 2 Division of Neurosurgery, Department 6–Clinical Neurosciences, Faculty of Medicine, “Carol Davila” University of Medicine and Pharmacy, 020021 Bucharest, Romania; 3 Clinic of Neurosurgery, “Dr. Bagdasar-Arseni” Emergency Clinical Hospital, 041915 Bucharest, Romania; 4 Department 11 of Plastic and Reconstructive Surgery and Pediatric Surgery, Faculty of Medicine, “Carol Davila” University of Medicine and Pharmacy, 020021 Bucharest, Romania

**Keywords:** jugular veins, computed tomography, neck veins, fenestration, duplication

## Abstract

(1) *Background*: The external jugular vein (EJV) descends on the sternocleidomastoid muscle to drain deep into the subclavian vein. Anatomical variations of the EJV are relevant for identification of the greater auricular nerve, flap design and preparation, or EJV cannulation. (2) *Methods*: Different publications were comprehensively reviewed. Dissections and three-dimensional volume renderings of peculiar cases were used to sample the review. (3) *Results*: Different anatomical possibilities of the EJV were critically reviewed and documented: fenestrations and double fenestrations, true or false duplications, triplication, absence, aberrant origin or course, or bifurcation. Tributaries of the EJV, such as the facial and posterior external jugular veins, are discussed. The internal jugular vein termination of the EJV is also presented. (4) *Conclusions*: Care should be taken when different morphological features of the EJV are encountered or reported.

## 1. Introduction

The external jugular vein (EJV) descends superficially to the sternocleidomastoid muscle and drains into the subclavian vein after penetrating the deep cervical fascia [1]. Along the EJV lie the lymph nodes of the superficial external jugular chain [2]. The current review aims to thoroughly analyze the anatomical variations of the EJV available in publications. A scientific review should not only look over multiple studies and briefly present them but also remark on their merits or even drawbacks. The variable morphology of the EJV should be deeply understood to further the improvements in the area of neck surgery. With its numerous natures, the EJV may adopt unexpected topographical positions or even morphological patterns a surgeon should be aware of. If these aspects are not well known, various complications may occur throughout the surgery. Iatrogenic vascular injuries often lead to severe outcomes or even mortality. For an improved view of the anatomical and clinical features of the EJV, different publications on this topic were reviewed. There were no exclusion criteria and all the studies that were related to human anatomical variations of the EJV were assessed. Moreover, we used archived computed tomography angiograms from previous studies [3,4,5] as well as dissections to support this review.

## 2. Origin and Drainage of the External Jugular Vein

The anatomical standard description of the EJV presents it as a superficial vein of the neck, usually formed by the union of the posterior division of the retromandibular vein (RMV) with the posterior auricular vein (PAV) [6,7,8,9,10,11,12].

Even though the regular formation of the EJV is commonly accepted in its unique form, its termination has been differently described by various anatomists. Thus, along with subclavian vein drainage, other EJV terminations might also be encountered: jugular-subclavian confluence and the internal jugular vein (IJV) [13]. Two pairs of valves were found in the EJV, one pair at its termination and the other a little above it [6]. 

Anatomical variations of neck veins could be better understood after observing the main events during embryogenesis. In 10 mm embryos, the primitive maxillary vein drains into the precardinal vein, and the ventral pharyngeal vein drains initially into the common cardinal vein and, later, into the precardinal vein [6]. Further, in 18 mm embryos, the ventral pharyngeal vein becomes linguofacial vein into which the RMV starts to drain [6]. The EJV arises caudally, from the cranial part of the ring the cephalic vein forms around the clavicle [6]. In this stage, the primitive maxillary vein anastomoses with the linguofacial vein [6]. In 40 mm embryos, the EJV connects cranially with the RMV by a posterior connection, and with the facial vein by an anterior connection [6]. This anterior connection degenerates until birth, and the facial vein remains connected only to the IJV [6]. The RMV remains connected to the IJV system by its anterior division and with the EJV by its posterior division [6]. Therefore, a persistent anterior connection of the primitive EJV would determine different connections between the EJV and facial vein.

### 2.1. Topography of the External Jugular Vein

#### 2.1.1. Relations of External Jugular Vein with Cervical Nerves

**Figure 1:**
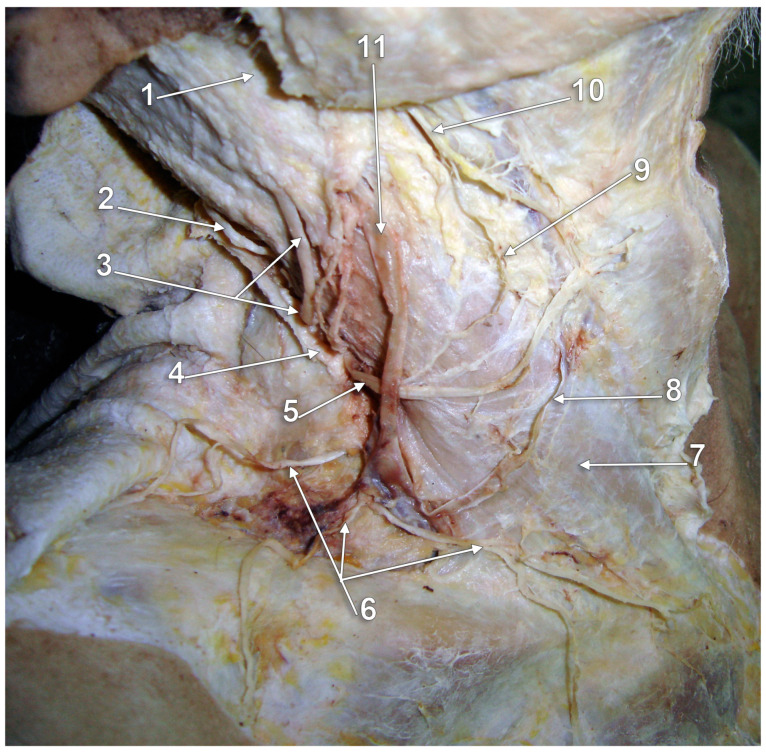
Right external jugular vein (EJV). Right side dissection, lateral view. 1. parotid gland; 2. lesser occipital nerve; 3. greater auricular nerve; 4. *punctum nervosum*; 5. transverse cervical nerve; 6. supraclavicular nerves; 7. sternocleidomastoid muscle; 8. communicating vein; 9. superficial cervical ansa; 10. cervical branch of the facial nerve; 11. EJV.

The upper segment of the EJV is closely related to the greater auricular nerve (GAN) (Figure 1). The length of the bundle formed by the two is individually variable, depending on the level of the emergence of the GAN along the posterior aspect of the sternocleidomastoid muscle (SCM). Multiple trunks of the GAN could be encountered (Figure 1). Transposition of the EJV and the GAN result in an anteriorly placed GAN [14].

Even though the EJV coursing along the posterior margin of the SCM faces great variability, the GAN may usually be found at approximately 1 cm posterior to the EJV (measured at 4 cm below the tip of the mastoid process) [14]. Dissections performed in this area should be carefully made, taking into account the proximity between the two. The GAN’s course in the immediate proximity of EJV should be noted. While both the nerve and the vein run superficial to the SCM, the GAN adopts an intrafascial course through the investing layer of the SCM. Iatrogenic injury may be brought not only to the EJV but also to the GAN, leading to paresthesia in its territory: numbness of the posterior-inferior ear lobe and preauricular and postauricular skin [15].

The topographical difference between the GAN point (*punctum nervosum*) and Erb’s point should be made. The latter one is not the anatomical spot at which the GAN may be found as it is a “supraclavicular point” located superior to the clavicle. Confusion between the two points should be avoided [16,17].

#### 2.1.2. Relation of the External Jugular Vein to Neck Muscles

The EJV courses close to several muscles of the neck. Its upper segment descends superficial to and obliquely across the SCM, then places itself at the posterior side of the muscle. The EJV continues its descent anterior to the anterior scalene muscle before joining the subclavian vein deep in the SCM [18].

The platysma muscle may entirely cover the superficial aspect of the EJV. However, consistent variations may occur. The EJV could be covered by the platysma muscle throughout its extension, or it could border the postero-superior muscle fibers or could course posterior to the platysma muscle at a maximum distance of 3 cm [8]. The free edge of the platysma muscle was found most often posterior to the EJV [14]. The EJV should be carefully identified at the posterior-superior border of the platysma muscle. Different flaps are used for intraoral reconstructions after cancer resection [19]. The platysma myocutaneous flap is suitable for small defects [19]. The IJV drains the flap anteriorly, while the EJV drains it posteriorly [20].

#### 2.1.3. Relation of the External Jugular Vein with Lymph Nodes

Lymph nodes are mainly linked to the venous system, and it is often not clear whether there is an arterial supply at all [21]. As quoted in [21], Visconti et al. (2017) proposed a novel concept pertaining to the venous lymph node flap—a vein with the surrounding lymph nodes could be transplanted to a major vein of a lymphedematous tissue [22]. Visconti’s experiments used venous lymph node flaps based on the EJV [22].

### 2.2. Morphology of the External Jugular Vein

The EJV is extensively variable in size and shape. It may be visible on the lateral aspect of the neck. The mean EJV diameter is 9.3 mm [23]. There may be an inverse correlation between the EJV diameter and that of the IJV [23]. EJV duplications or fenestrations may occur and will be detailed further in this paper.

### 2.3. The External Jugular Vein’s Tributaries

Commonly, the EJV has four main tributaries [13]: (1) anterior jugular vein (AJV) in 46% of cases; (2) transverse cervical vein in 88% of cases; (3) suprascapular vein in 47% of cases; (4) cephalic vein in 2% of cases, and in 11 out of 100 cases, the cephalic vein drains into the axillary vein and partially into the EJV through a superficial anastomosis.

**Figure 2:**
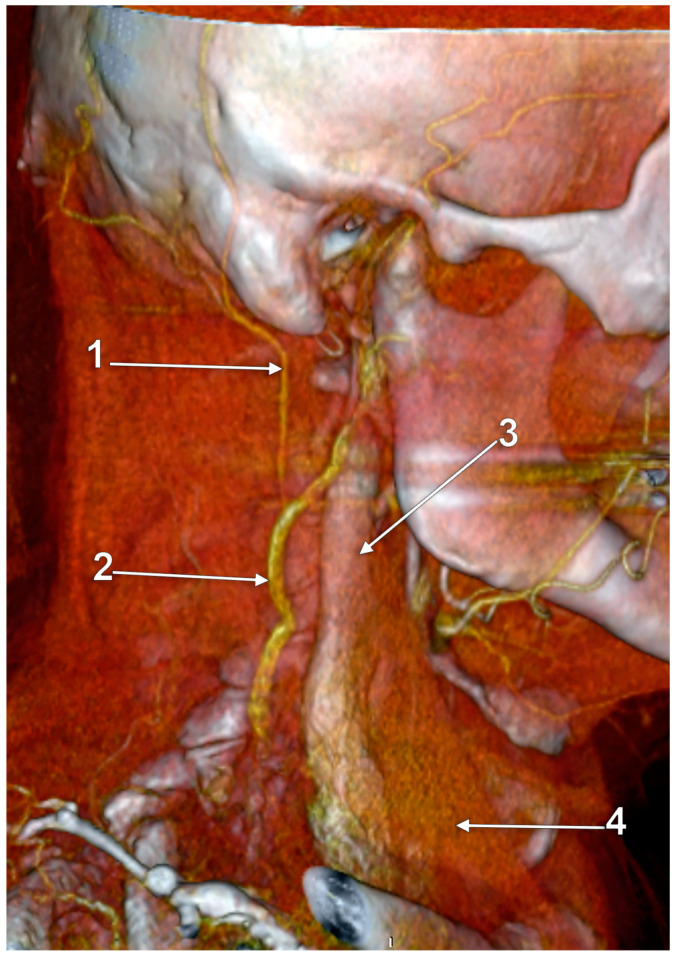
Right external jugular vein (EJV). Three-dimensional volume rendering (3D-VR). Right lateral view. 1. occipital vein; 2. EJV; 3. internal jugular vein; 4. sternocleidomastoid m.

**Figure 3:**
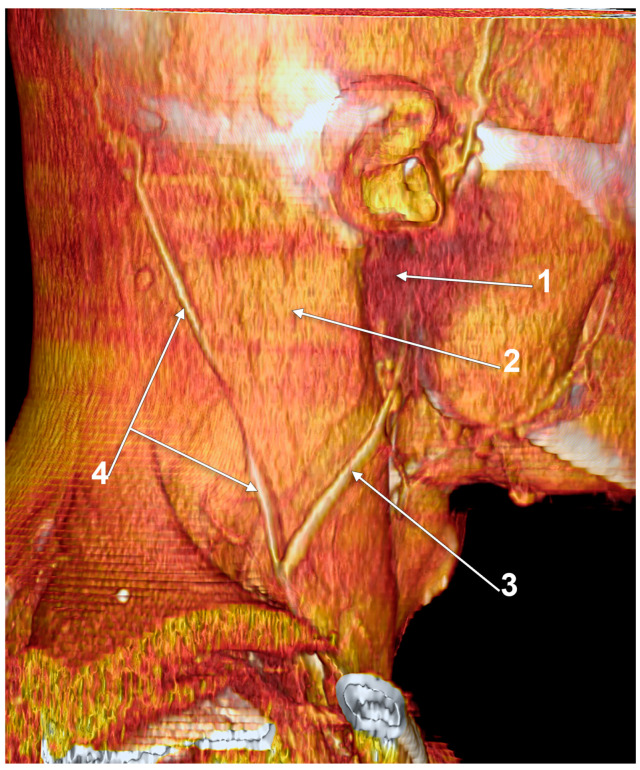
Posterior external jugular vein coursing along the posterior border of the sternocleidomastoid muscle (3D-VR). Right lateral view. 1. parotid gland; 2. sternocleidomastoid m.; 3. external jugular vein; 4. posterior external jugular vein.

**Figure 4:**
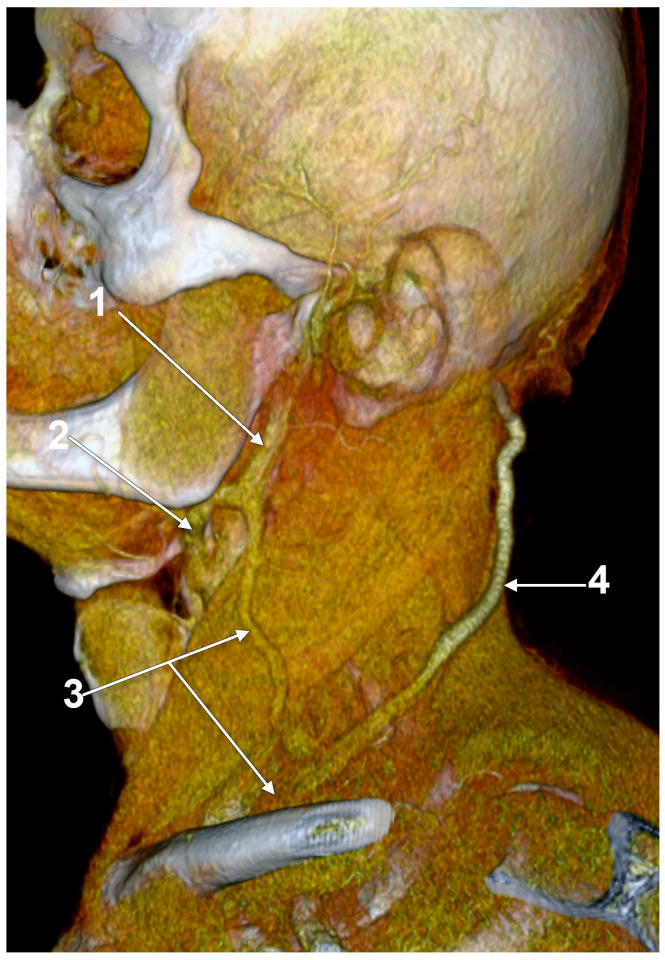
Posterior external jugular vein (PEJV) distanced from the sternocleidomastoid muscle (3D-VR). Left lateral view. 1. retromandibular vein; 2. anterior branch of the retromandibular vein; 3. external jugular vein; 4. PEJV.

**Figure 5:**
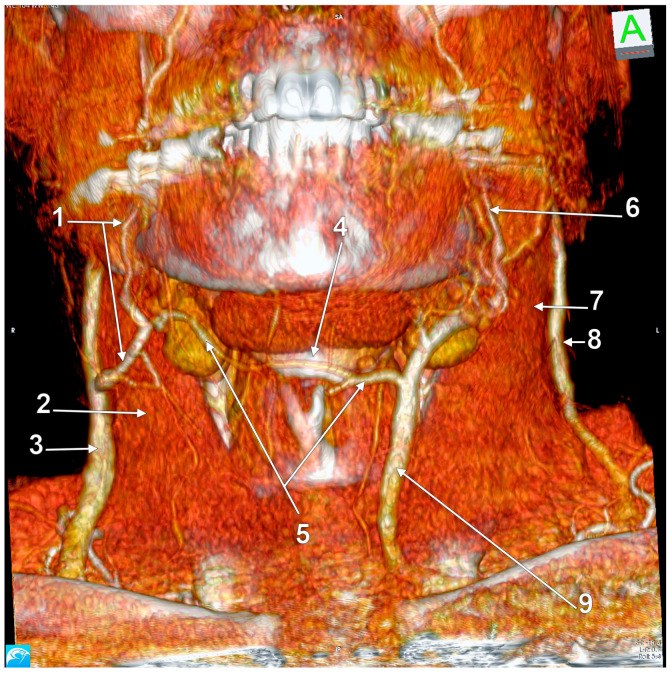
Right external jugular vein (EJV) anastomosis with the left anterior jugular vein (AJV). Absent right AJV; 3D-VR, anterior view. 1. right facial vein; 2. right sternocleidomastoid muscle; 3. right EJV; 4. hyoid bone; 5. transverse hyoid anastomosis of the right EJV and left AJV; 6. left facial vein; 7. left sternocleidomastoid muscle; 8. left EJV; 9. left AJV deriving from the facial vein.

**Figure 6:**
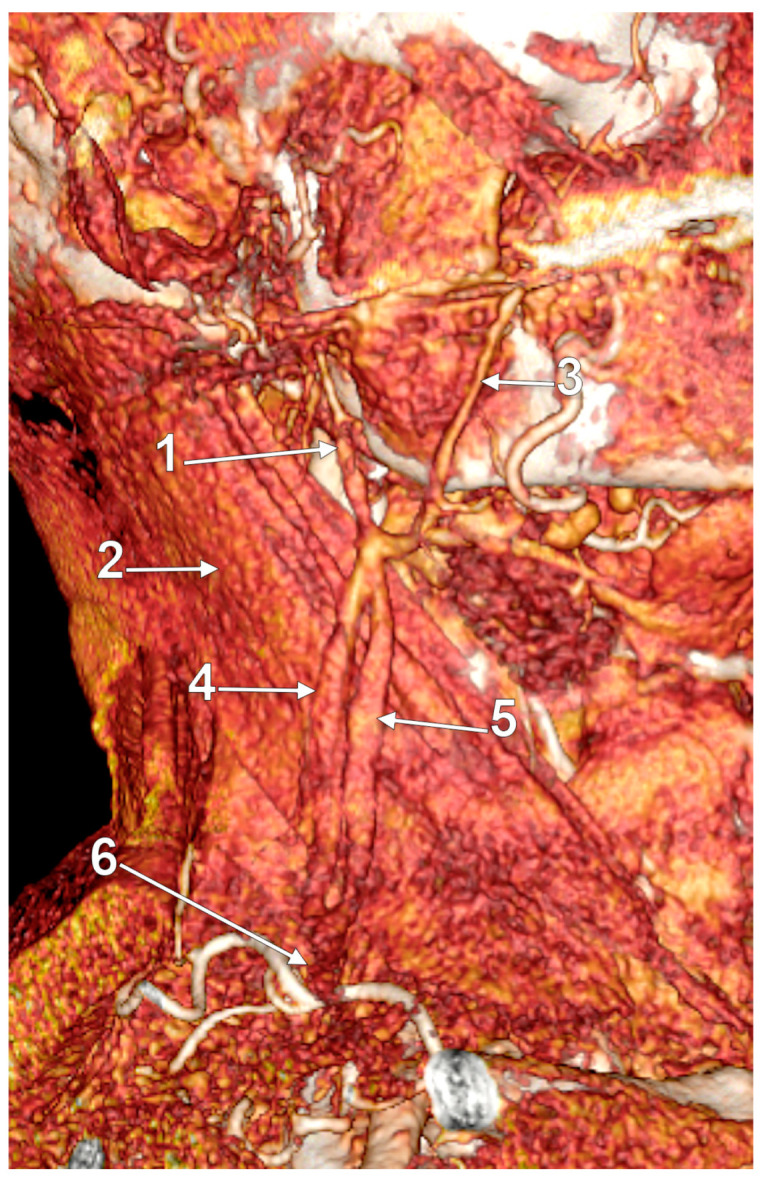
Large fenestration of the external jugular vein (EJV); 3D-VR, right lateral view. 1. undivided retromandibular vein; 2. sternocleidomastoid muscle; 3. facial vein; 4. posterior arm of the fenestrated EJV; 5. anterior arm of the fenestrated EJV; 6. common distal trunk of the EJV.

**Figure 7:**
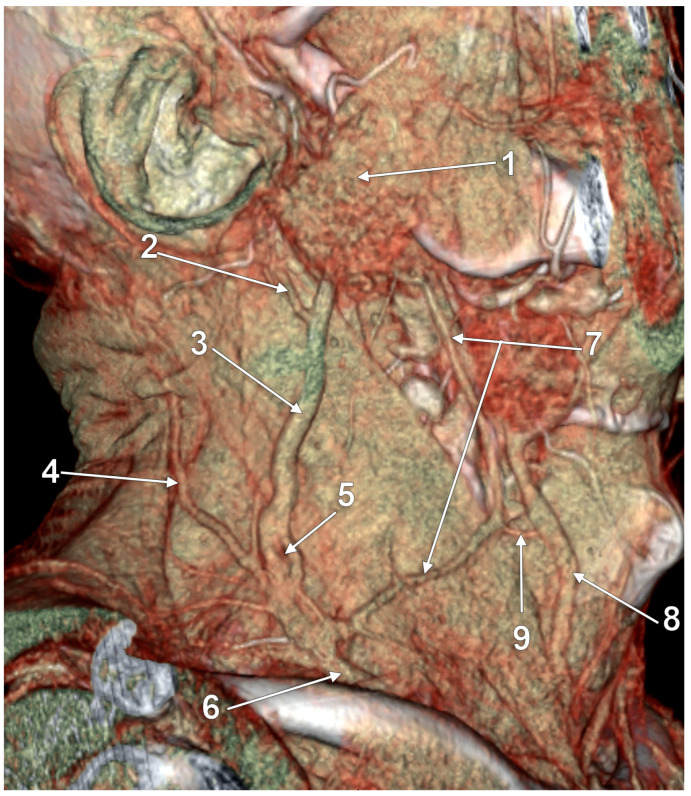
True duplication of the external jugular vein and fenestration of its posterior arm (3D-VR). Right lateral view. 1. parotid gland; 2. posterior auricular vein; 3. posterior arm of the duplicated external jugular vein (PEJV); 4. posterior external jugular vein; 5. fenestration of the PEJV; 6. common inferior trunk of the external jugular vein; 7. anterior arm of the duplicated external jugular vein (AEJV); 8. anterior jugular vein; 9. AEJV-to-anterior jugular communicating vein.

Other possible tributaries of the EJV are the occipital vein [24,25] (Figure 2), posterior external jugular vein (PEJV) (Figure 3 and Figure 4), facial vein [6,26,27,28], or linguofacial venous trunk [29]. In just 8% of cases the facial vein drains into the EJV [30] (Figure 5, Figure 6 and Figure 7). The facial vein could also drain into the IJV or the AJV [31].

Two main types of facial vein drainage into the EJV were described [26]. In type I, the facial vein drains into the EJV without cross connections, with varying degrees of obliquity in a Y-shaped, U-shaped, tuning-fork-shaped, or N-shaped (1 case, 6.2%) pattern [26]. In type II, the facial vein drains into the EJV with cross connections, either with an inverted A-shaped pattern or with a stepladder-shaped pattern [26].

Dalip et al. (2018) reviewed the vein of Kocher as the posterior external jugular vein (PEJV) originating in the occipital region and traveling “obliquely along the anterior border of the sternocleidomastoid muscle” to drain eventually into the common facial vein (FV), IJV, EJV, jugular venous arch, or even the brachiocephalic vein [32]. The PEJV was also described as running in the posterior aspect of the neck and usually draining into the middle segment of the EJV, not being assigned any eponym [33]. Therefore, the PEJV is a superficial vein of the lateral triangle of the neck. However, according to Bergman’s Encyclopedia of Human Anatomic Variation, the communicating vein, “also known as the vein of Kocher, is often a branch of the common facial vein” and usually drains into the anterior jugular vein [31]. The authors also document an additional thyroid vein as vein of Kocher or the “fourth thyroid vein” that “is found between the middle and inferior thyroid veins and drains into the internal jugular vein” [31], which seems the correct assignment of the eponym [34].

The PEJV is formed by numerous superficial veins of the neck or by the PAV if it does not join the RMV to form the EJV. The PEJV was mentioned in a report [35] but with no further information or quote regarding it. Frequently, it has been described as originating in the occipital region, traveling near the SCM muscle, and draining into the IJV/EJV/brachiocephalic vein [32]. In these regards, the PEJV could be regarded just as an occipital vein draining inferiorly into the EJV (Figure 3 and Figure 4). The evidence regarding this structure [12] is most often cited from anatomy atlases [36,37,38]. There is only one published case [39] where the PEJV is seen on CT and angiography and proves the existence of such a structure. However, in that particular case, the PEJV drained into the left subclavian vein instead of the EJV. Thus, considering the lack of evidence regarding the PEJV, specific literature should first establish whether or not this anatomical structure has any relation to the EJV.

## 3. Anastomoses of the External Jugular Vein

**Figure 8:**
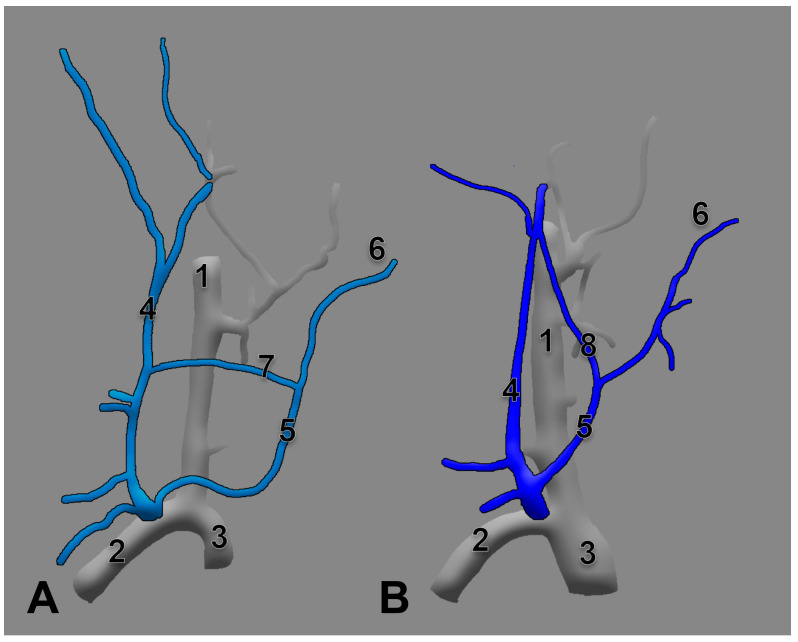
Anastomoses of the right external jugular and anterior jugular systems are depicted differently in Rouvière’s (**A**) and Pernkopf’s (**B**) drawings. Modified after [25,40]. 1. internal jugular vein; 2. subclavian vein; 3. brachiocephalic vein; 4. EJV; 5. AJV; 6. submental vein; 7. transverse anastomosis of the external jugular and anterior jugular veins; 8. oblique anastomosis of the retromandibular and anterior jugular veins.

A transverse anastomosis of the external and anterior jugular veins is nicely depicted in Rouvière and Delmas’ textbook [40] as well in Gray’s Anatomy [1], respectively. Interestingly, Pernkopf presents the AJV as formed by an anterior branch of the posterior division of the RMV, coursing obliquely at the anterior border of the SMC, and a submental vein descending over the hyoid bone [25]. The two different morphologies of the EJV are presented and compared in Figure 8.

**Figure 9:**
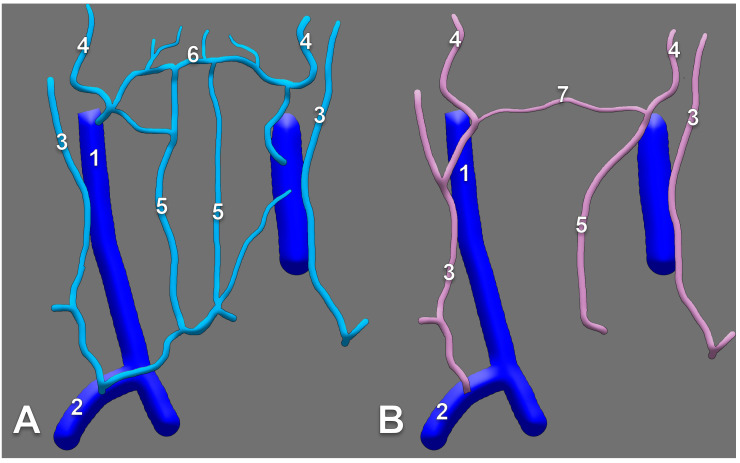
The suprahyoid anastomosis of facial veins ((**A**), modified from [38]), compared with (**B**), the transverse hyoid anastomosis of the right external and left anterior jugular systems in Figure 5). 1. internal jugular vein; 2. subclavian vein; 3. external jugular vein; 4. facial vein; 5. anterior jugular vein; 6. submental/suprahyoid anastomosis of the facial veins; 7. transverse hyoid anastomosis of the right external and left anterior jugular systems (anatomic variant).

The original drawing in Gray’s Anatomy indicate a transverse submental (suprahyoid) anastomosis of the proper facial veins draining each into the IJV [38]. However, when a facial vein drains into the EJV and the contralateral one continues as AJV, a transverse hyoid anastomosis of the two veins could result (Figure 5 and Figure 9).

## 4. Anatomical Variations of the External Jugular Vein

### 4.1. Duplication and Fenestration of the External Jugular Vein

**Figure 10:**
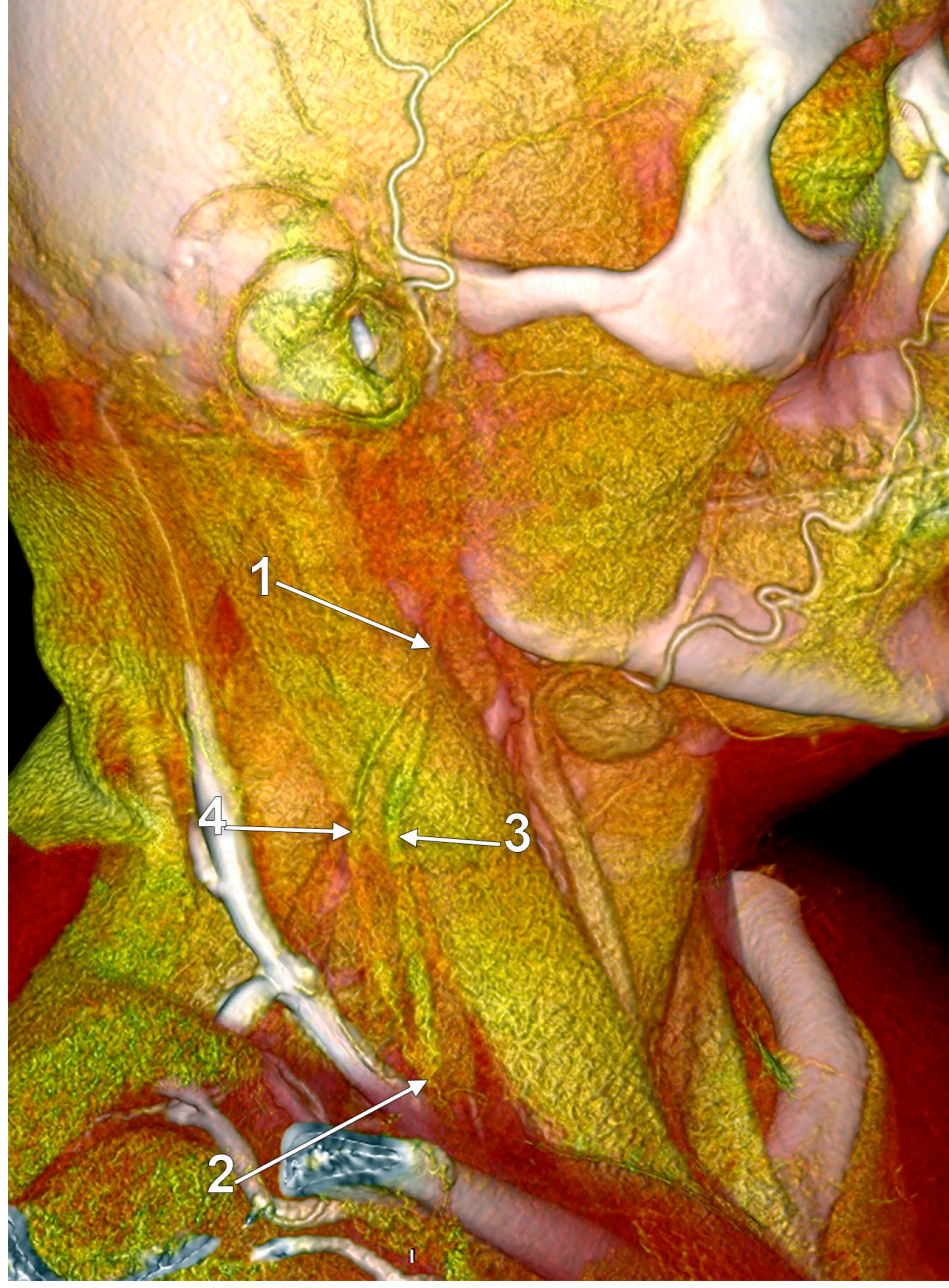
Large fenestration of the external jugular vein (EJV); 3D-VR, right lateral view. 1. proximal unique segment of the EJV; 2. distal unique segment of the EJV; 3. anterior arm of the EJV fenestration; 4. posterior arm of the EJV fenestration.

**Figure 11:**
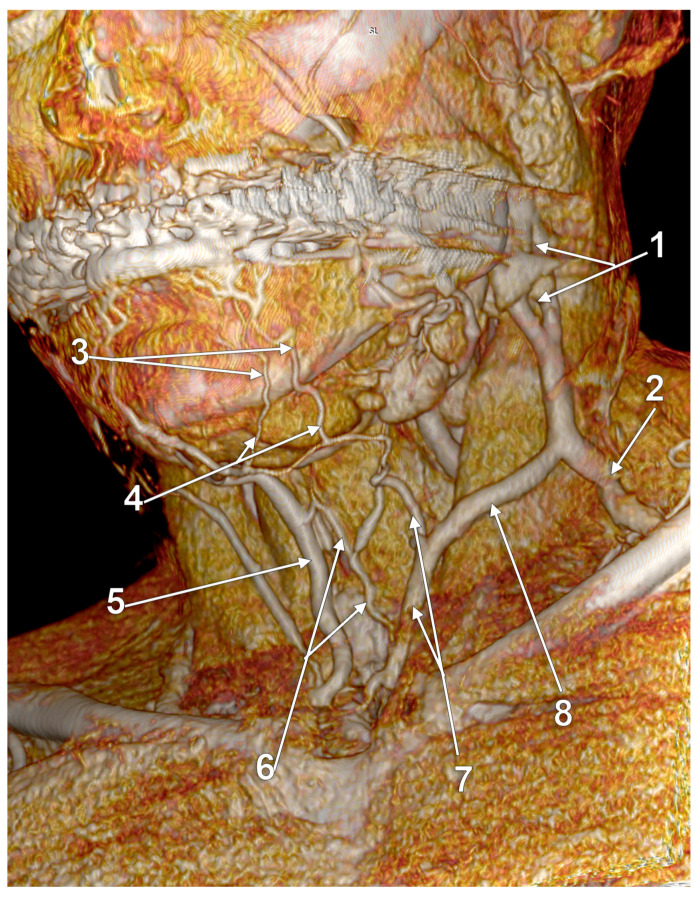
Fenestrated external jugular vein (3D-VR). Submental venous network draining the inferior labial veins. Anterior jugular network. Left antero-infero-lateral view. 1. proximal fenestration of the external jugular vein; 2. distal segment of the external jugular vein; 3. left inferior labial veins; 4. submental venous network; 5. right anterior jugular vein; 6. median cervical network; 7. left anterior jugular vein; 8. communicating vein.

**Figure 12:**
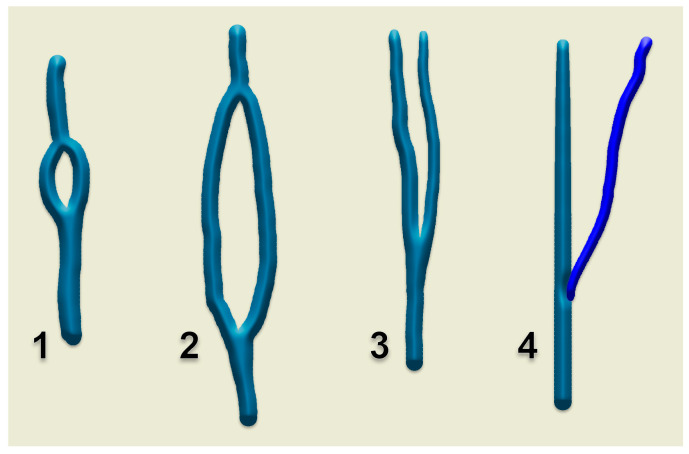
Diagrams of morphologic possibilities of EJV fenestration or duplication. 1. small, slit-like fenestration; 2. large fenestration; 3. true duplication; 4. false duplication.

**Figure 13:**
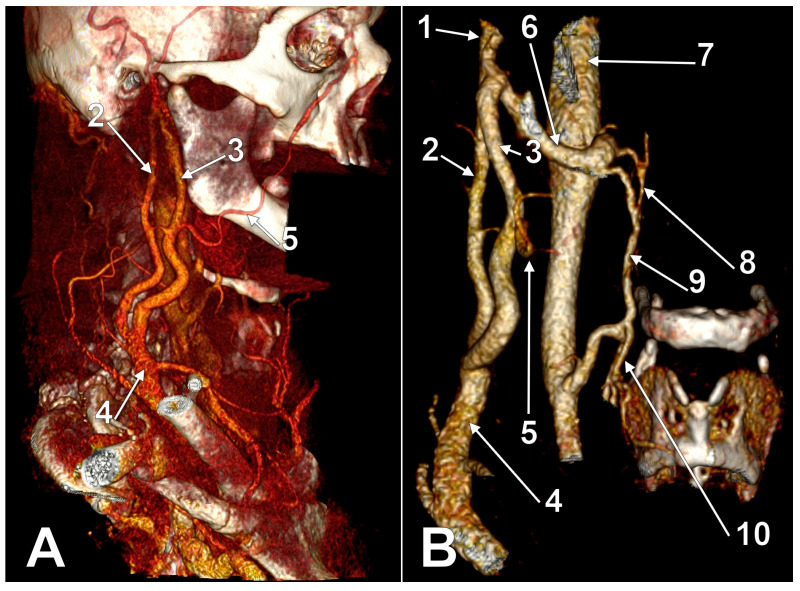
True duplication of the right external jugular vein. Trifurcated retromandibular vein. Prejugular anastomosis of the retromandibular and internal jugular veins (3D-VR). (**A**) Right lateral view. (**B**) Anterior view. 1. trifurcated retromandibular vein; 2. posterior arm of the duplicated external jugular vein; 3. anterior arm of the duplicated external jugular vein; 4. inferior common trunk of the external jugular vein; 5. facial vein; 6. anterior branch of the retromandibular vein (ARMV); 7. internal jugular vein (IJV); 8. superior thyroid vein; 9. prejugular ARMV-to-IJV anastomosis; 10. middle thyroid vein.

**Figure 14:**
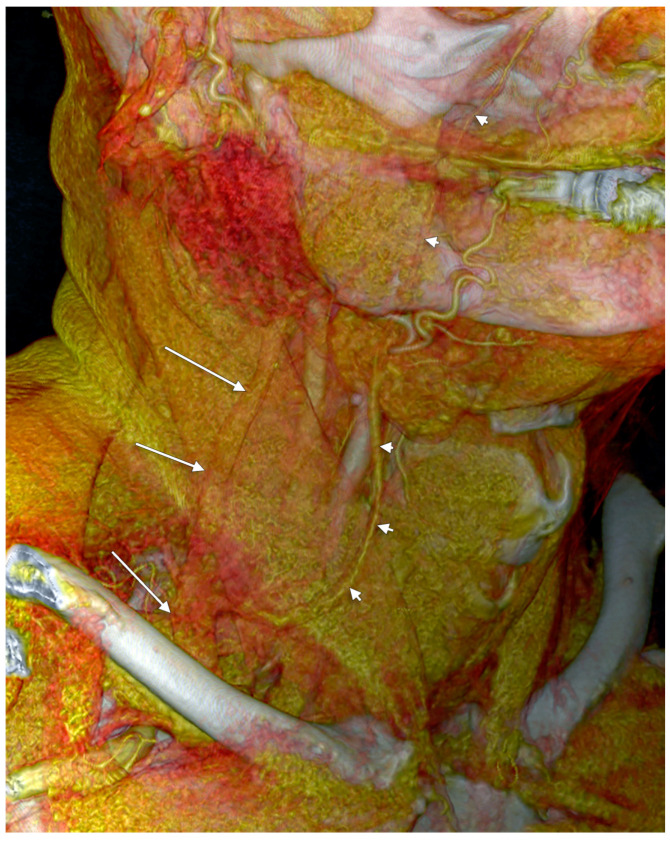
False duplication of the external jugular vein (3D-VR). Right antero-lateral view. The external jugular vein (arrows) descends on the sternocleidomastoid muscle. The facial vein (arrowheads) courses anteriorly and drains into the external jugular vein in the supraclavicular triangle.

First and foremost, clarification of each term should be undertaken to accurately classify each such case. Confusion has been generated in the literature due to erroneous usage of the two terms, sometimes even operated with the same meaning [41]. Thus, fenestration should be defined as a “window” (lat. fenestra = window) in the lumen of the vessel, a defined segment of the vessel where it divides into two branches following to reunite in the same vessel. Therefore the term “fenestration” indicates one origin and one end of the vessel (Figure 10, Figure 11 and Figure 12). Duplication, however, involves the bifurcation of the lumen without rejoining. Therefore, two separate blood vessels are formed and communication between the two exists at only one end [42,43]. The term “duplication” indicates two origins but one end [43] or vice-versa. For the EJV to be duplicated, the two arms of that duplication should have different origins, or ends, but in the same region (i.e., parotid region) (Figure 12 and Figure 13). If a different vein, with different topography, continues on the SCM to converge with the EJV, that variant should be regarded as a false duplication, being in fact an EJV receiving a tributary from a different region (Figure 12 and Figure 14). True duplications of the EJV could co-exist with fenestrations on the same side (Figure 7). Some authors used the syntagm “duplication/fenestration of the EJV” when reviewing the anatomic variations of the EJV [10]. Snoj and Cvetko (2013) found a two-in-one variant of the EJV: a large fenestration followed distally by a true duplication in which two arms left that EJV to drain separately in the subclavian vein [41].

#### 4.1.1. Fenestrations Reported as Duplicated External Jugular Veins

**Figure 15:**
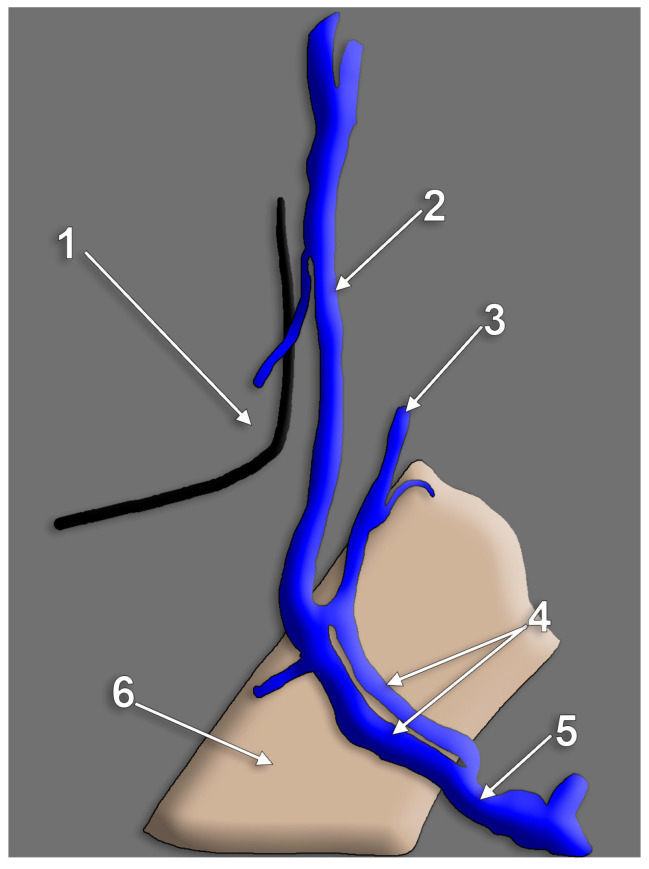
Diagram of a left side venous variant reported as “duplication of the external jugular vein”, drawn after. In the original dissection picture, only the sternocleidomastoid muscle and nerves were labeled. 1. angle of mandible; 2. undivided retromandibular vein; 3. posterior auricular vein; 4. upper fenestrated segment of the external jugular vein; 5. supraclavicular segment of the external jugular vein; 6. sternocleidomastoid muscle.

We documented previous reports of either “duplicated” or “fenestrated” EJVs. Different authors reported large fenestrations of the EJVs as duplicated EJVs [44,45,46], as resulting from the evidence they presented. Olabu et al. (2015) reported two successive fenestrations of the EJV as “multiple duplication” of that EJV [45]. Singla et al. (2011) reported the “partial duplication of the right EJV in the form of a venous ring enclosing supraclavicular nerve” [47]. To our opinion, a “venous ring” is a fenestration. Gerard III et al. (2021) reported as “duplicated” EJV a fenestrated EJV that was supplied superiorly by two unlabeled diverging veins (Figure 15) [48].

#### 4.1.2. Unidentified Duplications of the External Jugular Vein

Vollala et al. (2008) reported a case of “low formation” of EJV [27]. If that evidence is carefully observed, a false duplication of the EJV could be noticed with an anterior arm built-up by the facial vein coursing on the SCM, anterior to the EJV proper. A comparable morphology is presented in Figure 15.

#### 4.1.3. False Duplications of the External Jugular Vein Reported as True Ones

Shenoy et al. (2012) found a “double”, or “duplicated” EJV [11]. The posterior arm of that variant was an EJV continuing the RMV to end into the jugular-subclavian confluence. The anterior arm of that variant was a common facial vein coursing on the SMC, that further joined the anterior jugular vein to finally drain into the terminal part of the EJV. Therefore, a false duplication of the EJV was reported, with the two respective venous arms originating in different regions.

#### 4.1.4. Triplication of EJV

Even the “triplication” of EJV was reported [12]. Three veins were found by cadaver dissection on the SCM [12]. These were parallel and were regarded as medial, intermediate, and lateral EJVs [12]. However, the authors did not describe, or present convincing evidence regarding the end and eventual interconnections of those veins that could be as well a fenestrated or duplicated EJV coursing posteriorly to a variant facial vein draining distally into that EJV.

#### 4.1.5. External Jugular Vein’s Fenestration

Numerous cases of fenestration of the EJV have been published [49,50,51,52,53,54,55]. No specific length of the EJV until its fenestration was observed as it may occur at any anatomical spot, along the course of the vein. No accurate data was registered regarding the size of the fenestrations. Generally, the branches were referred to as “medial” and “lateral”. These could be rather regarded as anterior and posterior (Figure 6, Figure 7, Figure 10 and Figure 11). The relations of the rami with adjacent structures were not assessed. Cvetko (2013) reported a case in which the cervical branch of the facial nerve coursed through a fenestrated EJV to reach the punctum nervosum; the anterior arm of that fenestration was joined to the AJV by a transverse communicating vein [50]. Additionally, the transverse cervical and supraclavicular nerves were found coursing through fenestrations of EJVs [46,47,56,57]. However, EJV fenestrations could be as well empty [52].

Singh (2020) reported the anomalous formation of the EJV by anterior and posterior divisions of an RMV descending from the parotid and facial vein [9]. That venous variant could be equally regarded as a fenestrated EJV that continued an undivided RMV and was joined by the facial vein.

#### 4.1.6. Successive EJV Fenestrations/Double Fenestrated EJV

**Figure 16:**
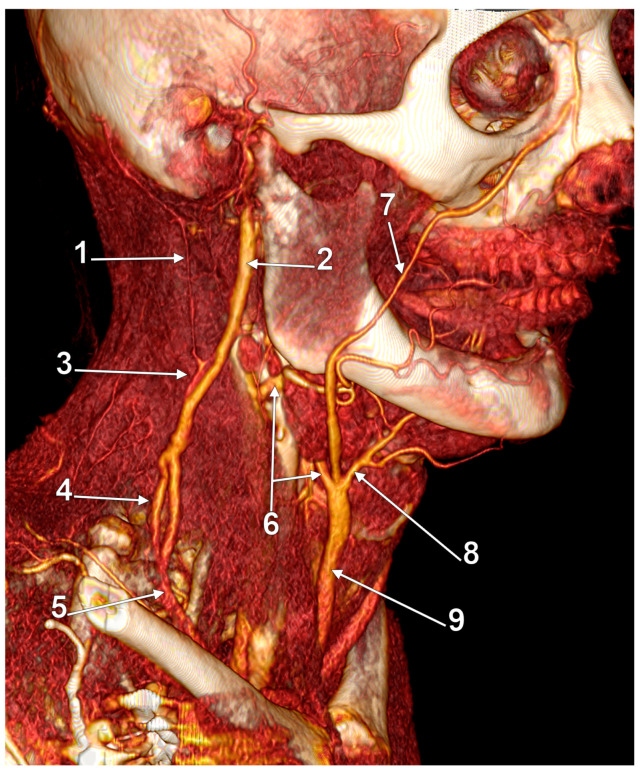
Double fenestration of the external jugular vein (EJV). Trifurcated origin of the anterior jugular vein; 3D-VR, right lateral view. 1. occipital vein; 2. undivided retromandibular vein; 3. proximal EJV fenestration; 4. distal EJV fenestration; 5. terminal end of EJV; 6. internal jugular-to-anterior jugular communicating vein; 7. facial vein proper; 8. submental vein; 9. anterior jugular vein.

Double-fenestrated EJVs could be also found with a proximal fenestration on the SCM and a distal, supraclavicular one (Figure 16).

#### 4.1.7. Retromandibular Vein’s Fenestrations

**Figure 17:**
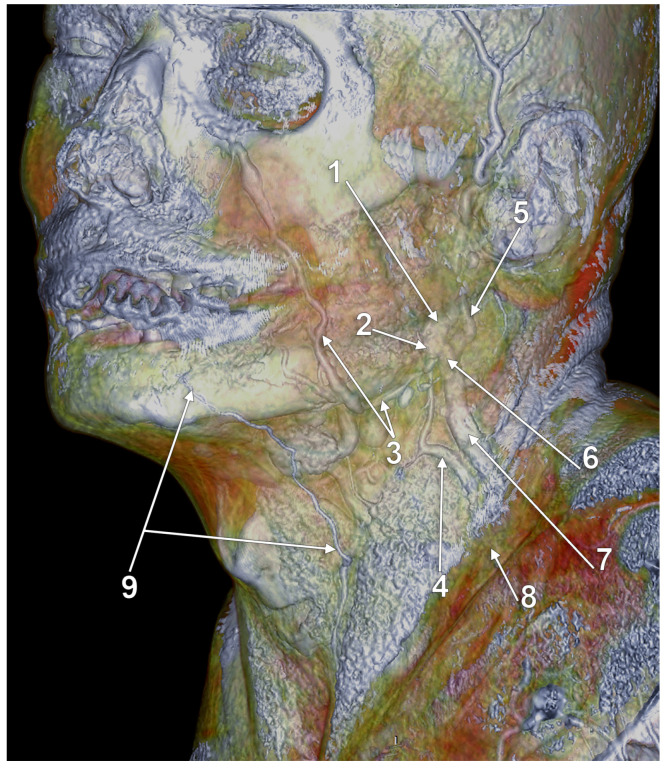
Duplicated retromandibular vein (RMV) continued as fenestrated external jugular vein (EJV); 3D-VR, left infero-lateral view. 1. RMV; 2. anterior division of the RMV; 3. facial vein proper; 4. anterior arm of the EJV fenestration, continuing (2); 5. posterior auricular vein; 6. posterior division of the RMV; 7. posterior arm of the EJV fenestration resulting from (6); 8. terminal end of the EJV; 9. inferior labial vein continued as anterior jugular vein.

**Figure 18:**
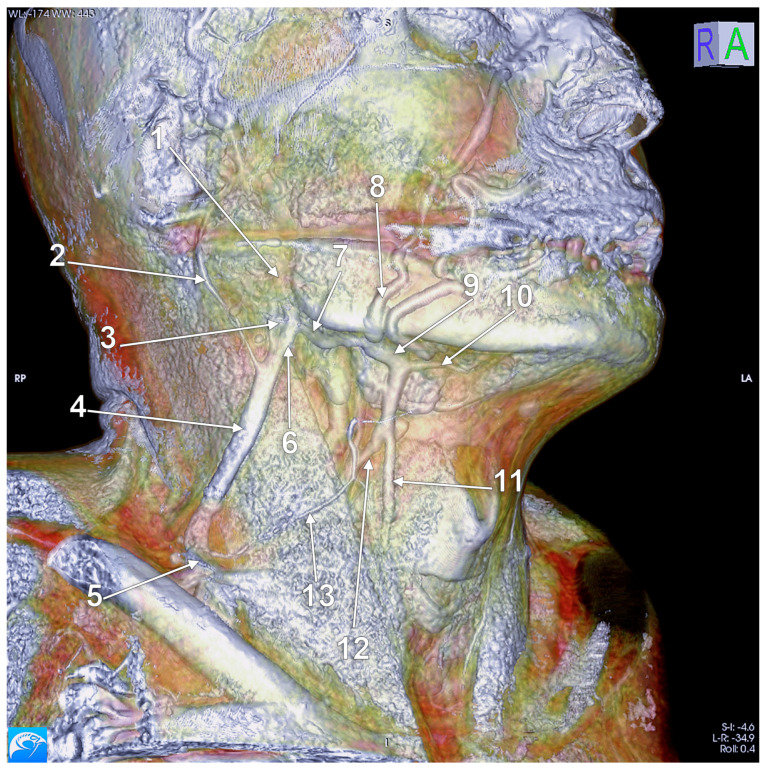
Fenestrated retromandibular vein (3D-VR). Right lateral view. 1. retromandibular vein; 2. long posterior auricular vein; 3. fenestrated retromandibular vein (RMV); 4. external jugular vein (EJV); 5. terminal end of the EJV; 6. posterior division of the RMV; 7. anterior division of the RMV; 8. facial vein proper; 9. common facial vein; 10. submental vein; 11. anterior jugular vein; 12. distal segment of the common facial vein draining into the internal jugular vein; 13. superficial anterior cervical vein draining over the sternocleidomastoid muscle to the EJV.

**Figure 19:**
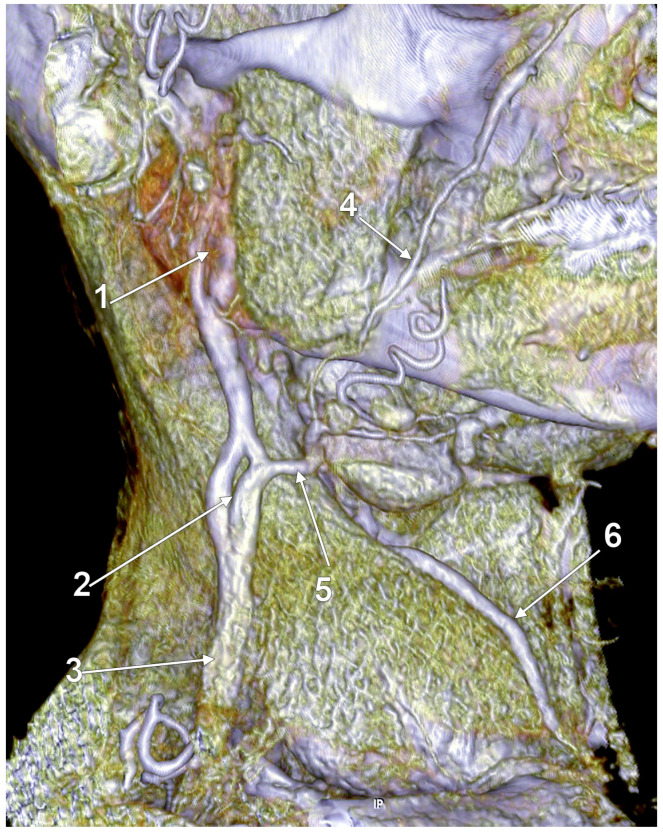
Fenestrated undivided retromandibular vein (RMV). Fenestrated external jugular vein (EJV); 3D-VR, right lateral view. 1. RMV fenestration; 2. EJV fenestration; 3. distal EJV; 4. facial vein proper; 5. internal jugular-to-EJV communicating vein appears as a substitute of the anterior division of the RMV; 6. anterior jugular vein.

According to Ponnambalam and Karuppiah, fenestrations of the EJV could result after both divisions of the RMV descend and reunite on the SCM [51], such as we present in Figure 17. However, we present in Figure 18 an RMV with both anterior and posterior divisions and with a fenestration of its main trunk and posterior division, further receiving the posterior auricular vein and continued as EJV. Another morphological possibility is that of successive RMV and EJV fenestrations (Figure 19) and the anomalous connection of the EJV fenestration, instead of that of the RMV, with the IJV.

#### 4.1.8. Bifurcated External Jugular Vein

Rao et al. (2018) reported a case of “bifurcation” of the EJV [58]. The respective authors describe the venous pattern as follows: “[…] two parallel veins were identified in superficial fascia of the neck. Both originated from a common trunk within the deep lobe of parotid gland and followed a course lateral to sternocleidomastoid and joined as a single vein of large caliber before penetrating deep fascia inferiorly” [58]. It clearly results that the “bifurcated” EJV is a fenestrated one.

### 4.2. Complex Venous Design on the Sternocleidomastoid Muscle

**Figure 20:**
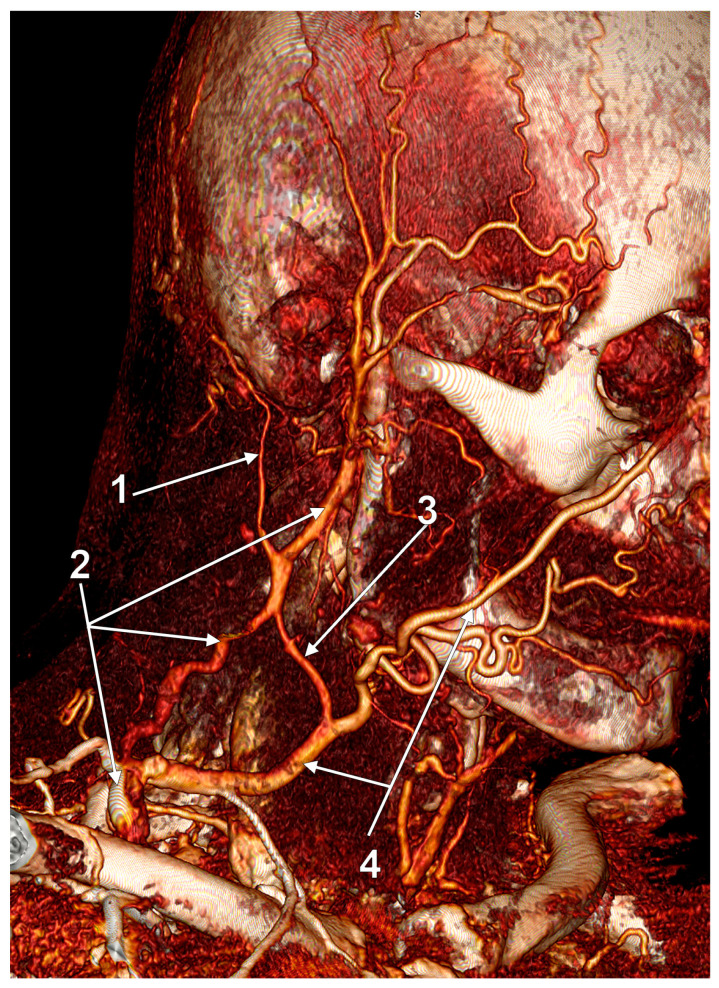
Complex venous architecture superficial to sternocleidomastoid muscle; 3D-VR, right antero-lateral view. 1. occipital vein; 2. external jugular vein; 3. communicating vein; 4. facial vein.

Anastomoses of the EJV with the facial and/or anterior jugular veins could determine a complex venous morphology on the SCM [45]. A complex venous architecture contributed by the EJV, facial vein, and occipital vein was found on the sternocleidomastoid muscle (Figure 20). The facial vein coursed on the SCM, antero-inferiorly to the EJV, to drain into it in the supraclavicular fossa. A communicating vein uniting proximally the EJV and facial vein modified that false duplicated pattern into a large venous triangle.

### 4.3. Absent External Jugular Vein

**Figure 21:**
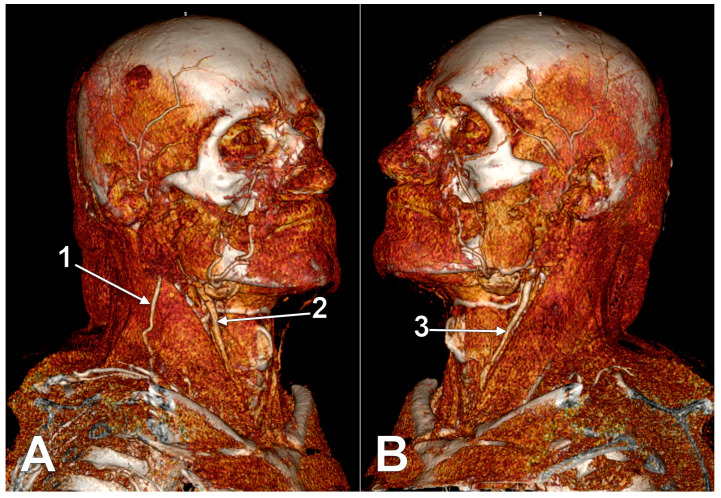
Absent left external jugular vein (3D-VR). (**A**) Right lateral view; (**B**) left lateral view. 1. right external jugular vein; 2. anterior branch of the right retromandibular vein continued as anterior jugular vein at the anterior border of the sternocleidomastoid muscle; 3. anterior branch of the left retromandibular vein continued as anterior jugular vein.

Undivided RMV (into anterior and posterior trunks) may lead to drainage of RMV into the IJV if there is an absent facial vein. An undivided RMV appears in less than 1% of cases (1 out of 104) [30]. If the facial vein drains into the undivided RMV, they form the common facial vein CFV, which then may terminate into the IJV [59]. In a male cadaver, the unilateral absence of the EJV was noted and seemingly resulted after an undivided RMV drained into the internal jugular system [60]. In a different study, the EJV was absent in 14.2% of cases, all males [45]. An absent EJV could be found if an undivided RMV continues as AJV (Figure 21).

### 4.4. Aberrant Origin or Course of the External Jugular Vein

**Figure 22:**
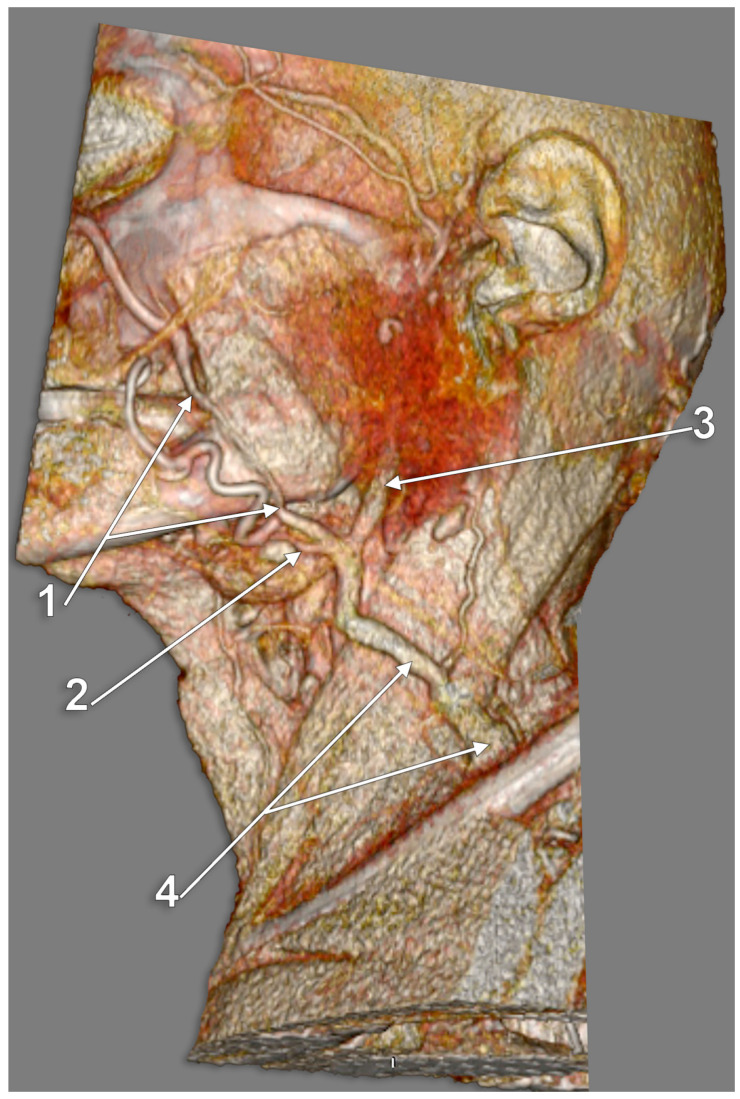
Facial vein continued as external jugular vein (3D-VR). Left side, infero-lateral view. 1. facial vein; 2. submental vein; 3. undivided retromandibular vein; 4. external jugular vein (variant).

The facial vein was found continuing on the SCM as EJV after receiving the RMV and submental vein [27]. A quite similar venous pattern is presented in Figure 22. Vani et al. reported recently a peculiar case of EJV formation [54]. As it results from the evidence these authors presented, the anterior division of the RMV left the parotid gland and united with the facial vein between the SCM and the IJV to further drain into the EJV [54]. Therefore, both divisions of the RMV supplied the EJV. It was also reported a dissection case in which the EJV continued posterior to the SCM but deep to the inferior belly of the omohyoid muscle to finally reach the subclavian vein [61].

### 4.5. Hypoplastic External Jugular Veins

**Figure 23:**
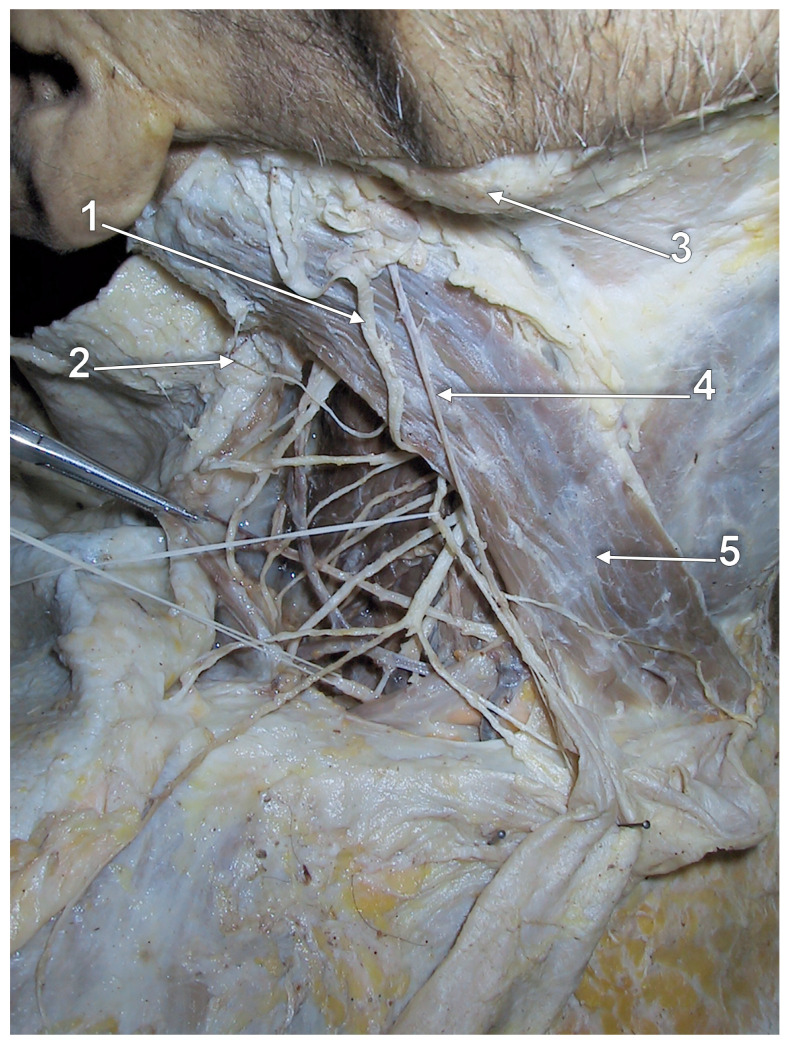
Hypoplastic external jugular vein. Cadaver dissection. Right side, lateral view. 1. great auricular nerve; 2. lesser occipital nerve; 3. angle of mandible; 4. hypoplastic external jugular vein; 5. sternocleidomastoid muscle.

In such cases, there is a normal formation of the EJV but with a hypoplastic morphology (Figure 23). The volume deficit could be taken over by a large anastomotic vein, which drains the common facial vein into the IJV/subclavian vein.

The specific literature currently faces a shortage of hypoplastic cases. There is not any classification in the literature regarding the specific size of a hypoplastic EJV. Thus, labeling a case of EJV as hypoplastic is simply a subjective assumption. “Hypoplasia” has been widely used in the literature to describe veins with “very small diameter” [62] or “very small dimensions” [63]. Not only has the absence of specific measures led to misunderstandings but also the anatomical fact that each vein has a different diameter with individual variations. The specific literature should establish physiological variations for each type of vessel. Thereby, inconsistency within those limits would imply hypo/hyperplasia of the specific vessel. 

### 4.6. Various Topography of the External Jugular Vein

EJV may adopt various topographical positions regarding its origin, course, and drainage. The origin of the EJV is most often encountered beneath the parotid gland, ear lobe, or auricle, behind the angle of the mandible [64]. The classification of its rapport with the SCM has not yet been established as it faces great variability. The diagonal trajectory on the superficial aspect of the SCM is most often depicted in anatomical illustrations. However, any logical course of the EJV toward the SCM muscle should be taken into consideration when surgically approaching the EJV without a previous MRI scan of the patient.

### 4.7. Multiple External Jugular Veins

Double EJV was described as two independent vessels emerging from the parotid gland [65]. Even if the study was conducted on 96 subjects, the only evidence is through drawn schemes. Further research should be conducted on the topic, and, eventually, dissection images or even CTs/MRIs may be published.

### 4.8. Internal Jugular Vein Drainage of the External Jugular Vein

**Figure 24:**
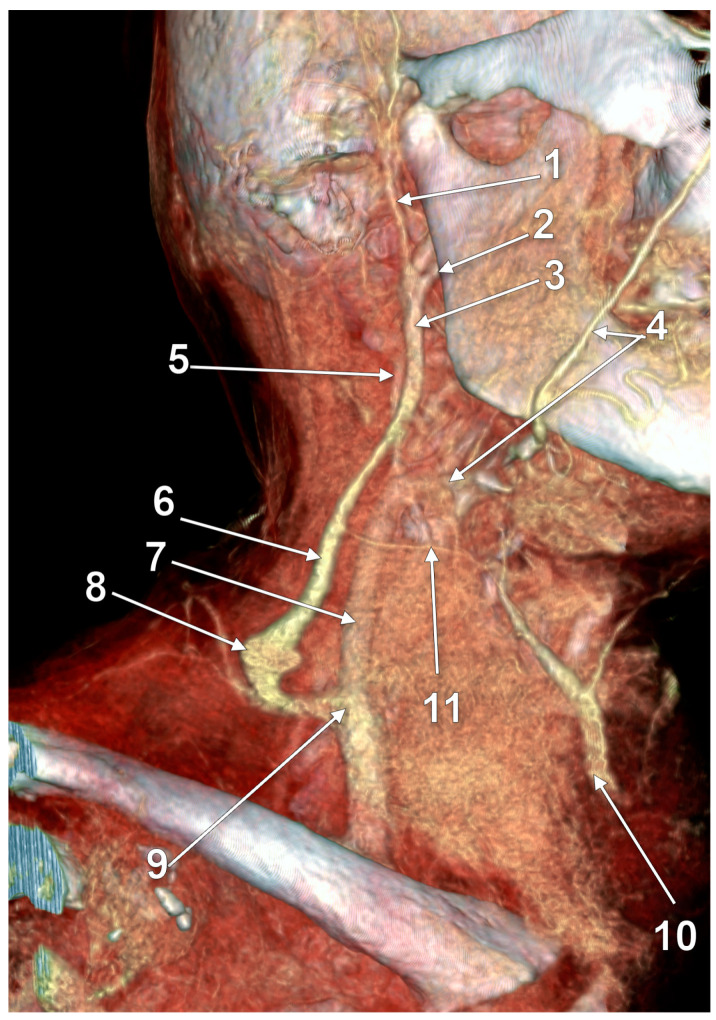
Internal jugular vein (IJV) drainage of an aneurysmal external jugular vein (EJV) via the Erb’s point (3D-VR). Right lateral view. 1. superficial temporal vein; 2. maxillary vein; 3. undivided retromandibular vein; 4. facial vein; 5. hypoplastic superior cervical segment of the IJV; 6. EJV; 7. normoplastic inferior cervical segment of the IJV; 8. EJV aneurysm; 9. EJV-to-IJV drainage; 10. anterior jugular vein (AJV); 11. EJV-AJV communicating vein.

The EJV drainage into the IJV was not documented previously. We present here such a rare variant (Figure 24). An undivided RMV continued as EJV and bent over the posterior border of the SCM to drain deep to it into the IJV. An aneurysm of that EJV was found at the posterior border of the SCM. In that case the facial vein drained into the IJV at the level of the hyoid bone. The IJV segment above that drainage was hypoplastic.

## 5. Clinical Applications

The EJV is an important drainage site for shunt procedures involving the lateral ventricle in hydrocephalus surgery, being also important for maxillo-facial surgery, percutaneous central vein cannulation, biocompatibility studies, total parenteral nutrition in critically ill patients, or invasive monitoring [6]. A cephalic vein to the EJV bypass becomes important in preserving the AV fistula for hemodialysis after subclavian vein thrombosis [6]. The cephalic vein and EJV are excellent alternatives for venous outflow in free breast reconstruction if neither the internal thoracic nor thoracodorsal veins are sufficient, especially in left-sided reconstructions [66].

### 5.1. External Jugular Vein Cannulation

Cannulation aids in conducting diagnostic procedures or intravenous therapies. It is a low-risk procedure due to the ultrasound guidance. External jugular veins may be accessed to sample the drainage of the cavernous sinus from the pituitary region in cases of Cushing’s disease (CD) with hypoplastic inferior petrosal sinus (IPS). Even if IPS sampling plays a significant role in the diagnostic evaluation of the CD [67], the hypoplasia of the IPS leads to a weak connection with the IJV. Therefore, due to the inadequate drainage of the cavernous sinus in the IPS, samplings of the IPS may become irrelevant and the EJV should be taken into account for endocrine venous sampling (cannulation, administration of corticotropin-releasing factor and blood collection) [68].

### 5.2. External Jugular Vein Phlebectasia

EJV phlebectasia represents a painless swelling or a neck mass of benign nature, which can be treated through surgical resection. The preferred method of investigation is through the Valsalva maneuver as it is an accurate, non-invasive technique adopted to distinguish the jugular enlargement. The above-mentioned procedure is described as a forced expiration against a closed glottis [69]. The difference between phlebectasia and varicosity should be made as the latter implies tortuosity without dilatation [70].

### 5.3. External Jugular Vein Aneurysm

Venous aneurysms are rare lesions, being previously reported in different anatomic locations [71]. They can be classified into primary (congenital) and acquired, the former being true aneurysms because they have an intact venous wall [71].

It occurs due to dilatation of the EJV at least 1.5 times compared with its initial diameter (Figure 24). It produces swelling of the neck which increases with the Valsalva maneuver and is usually painless [72]. In a pediatric case, an EJV aneurysm was barely visible at rest but became more prominent with crying [71]. The exact incidence of EJV aneurysm remains controversial [73].

The standard approach is represented by excision of the lesion after ligation of feeding vessels [71]. Direct puncture of the EJV may be another approach to such an aneurysm, especially if it is treated for aesthetic reasons (endovascular treatment) [74]. The external jugular vein system rate of thrombosis has not been studied yet, whereas in IJV it was up to 30% after neck dissection [75,76]. However, few cases of thrombosed aneurysms of the EJV were reported [77,78,79,80]. A thrombosed EJV aneurysm mimics a branchial cyst [73]. Such EJV aneurysms should be regarded as a possible source of pulmonary emboli [79].

### 5.4. External Jugular Vein Phlebectasia and Aneurysm

Jugular venous aneurysm or jugular phlebectasia is the most commonly encountered venous malformation involving the neck veins [74]. These two terms are often used to describe identical pathologies. Even though, no established classification can be found regarding venous malformations, and the terminology must be differentiated, as various vascular pathologies may be referred to [81]. Phlebectasia is often a congenital disease that occurs due to congenital weakness of the muscular layer of the wall and is considered a childhood disease. Aneurysm refers to a degenerative change of the venous muscular layer, which will form a saccular dilatation. It is usually encountered in adults after a traumatic lesion of the region and has a predilection for thrombosis [82,83]. 

Resection of such EJV pathologic formations should be limited to centers being aware of the risks of surgical resection of these variable cervical venous formations, and the indication for any surgical resection should be evaluated with caution since the risk of severe or even fatal bleeding, although rare, can be relevant.

## 6. Conclusions

The specific literature should properly use anatomical terms in order to describe the concerned topic. Generalization of topics with such variety as the EJV should not occur. Attempts to create a general classification so as to classify each case of EJV do not represent a viable option due to individual variability. Each case, especially those that demand a surgical intervention in the neck area, should be assessed with the support of medical imaging, such as MRIs and CTs.

## Data Availability

Data are available from authors on reasonable request.
